# The Study of a Novel Sorafenib Derivative HLC-080 as an Antitumor Agent

**DOI:** 10.1371/journal.pone.0101889

**Published:** 2014-07-08

**Authors:** Ke Tang, Can Luo, Yan Li, Chenshu Lu, Wanqi Zhou, Haihong Huang, Xiaoguang Chen

**Affiliations:** State Key Laboratory of Bioactive Substances and Functions of Nature Medicines, Institute of Materia Medica, Chinese Academy of Medical Sciences & Peking Union Medical College, Beijing, China; H. Lee Moffitt Cancer Center & Research Institute, United States of America

## Abstract

In this study, our objective is to evaluate the potential of a novel Sorafenib derivative, named HLC-080, as a new anticancer agent for colon cancer. We firstly carried out MTT assay, colony formation assay, flow cytometry analysis and transwell invasion assay to determine effect of our compound HLC-080 on cell viability, anti-proliferation activity, cell cycle arrest and the intervention on cell invasion, respectively. On the other hand, *in vivo* antitumor activity of HLC-080 was also tested using H22 xenograft model and the angiogenesis effect of HLC-080 was measured by EA.hy926 tube formation assay. The expression levels of various proteins in HLC-080 treated with HT-29 cell lines were examined using Western blot and ELISA experiments. The results showed that HLC-080 could dramatically inhibit the growth and colony formation of various tumor cells, therefore exhibited remarkable antitumor activity. HLC-080 can induce cell cycle arrest at G1 phase in HT-29 cells and subsequently inhibit the invasive potential of colon cancer cells. HLC-080 also exhibits anti-angiogenesis effect in EA.hy926 model. Additionally, the *in vivo* study showed that HLC-080 was able to reduced the tumor weight with the rate of 35.81%. And at the concentration of 0.352±0.034 µM, HLC-080 is able to reduce half of the regular protein level of p-c-Raf (Ser259), consequently block Raf/MEK/ERK signaling in HT-29 cell lines. In conclusion, our study suggests that Sorafenib derivative HLC-080 has the potential to inhibit cell proliferation and angiogenesis, Since, HLC-080 is particularly active against human colon cancer cells, our study highlights that HLC-080 and its related analogues may serve as a new anti-cancer drug, particularly against colon cancer.

## Introduction

Colon cancer is reported as the third highest incidence and mortality among all types of cancers in the western world [1], [2]. In China, colon cancer is ranked 3rd in mortality amongst all cancers. [3]. Rencently, due to the change of the dietary habits and lifestyle of the Chinese people, the rate of colon cancer has increased rapidly [4]. However, the treatment of colon cancer is challenging and little process in colon cancer therapy was developed over the past decades [5]. Chemotherapy of colon cancer still relies on a few general front-line anticancer drugs. However it was hard for these drugs to reach a satisfactory result [6], [7]. Certain cytotoxic agents, such as 5-Fluorouracil (5-FU) and capecitabine, were used as an adjuvant agent in the combination therapy of colon cancer [8]. Other commercial anticancer drugs that target vascular endothelial growth factor (VEGF) (Bevacizumab) and epidermal growth factor receptor (EFGR) (Cetuximab) also show little benefit to metastatic colon cancer. Furthermore, the treatment of bevacizumab and cetuximab even shows the trends towards worse outcomes [8]. Therefore, there is an urgent need to develop more effective and specific anti-colon cancer drugs. Recent studies shows that the inhibitors of PI3 kinase, c-Raf or other signaling pathways are effective against colon cancer cells, and hence shows the potential of being clinical anti-colon cancer drugs [5], [6], [8].

**Figure 1:**
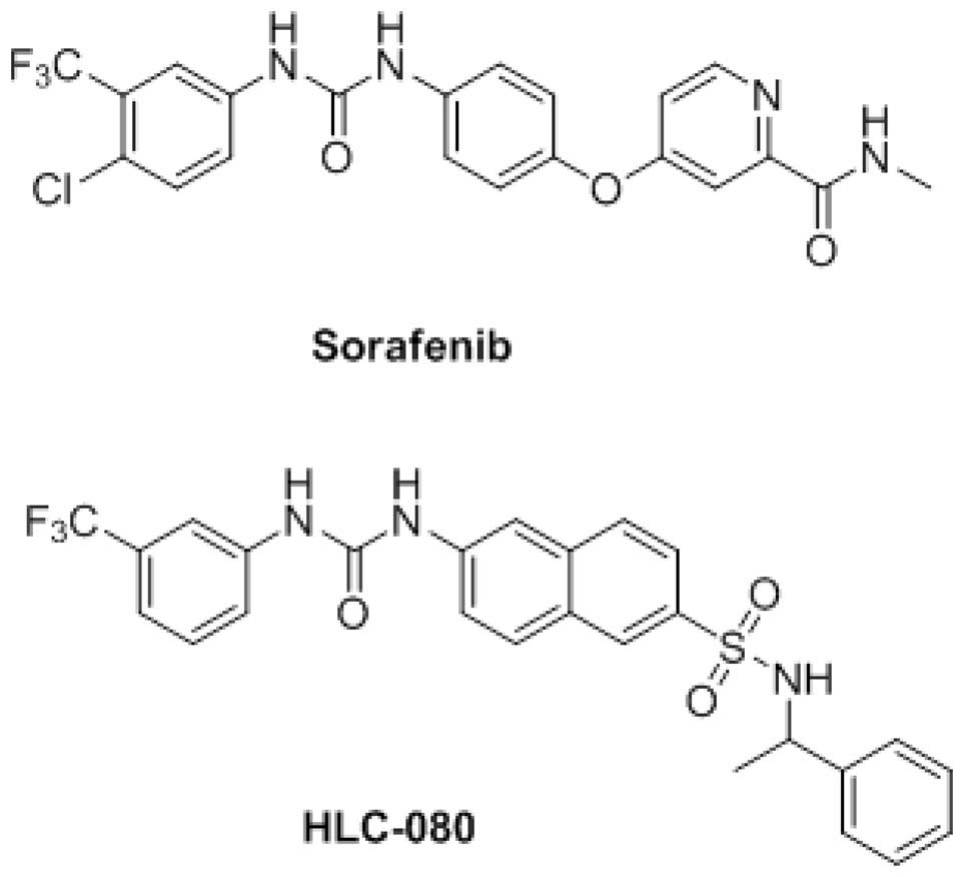
The chemical structure of Sorafenib and HLC-080.

Sorafenib (Nexavar) (Figure 1) is an oral anti-cancer drug that targets multiple kinases. Previous study showed that Sorafenib could blocks the growth of solid tumors mostly through the interruption of the Ras/Raf/MEK/ERK signaling cascade [9]–[14]. Moreover, it is also reported that Sorafenib targets several other receptor tyrosine kinases, including c-Raf, vascular endothelial growth factor receptor2 (VEGFR2), platelet-derived growth factor receptor (PDGFR), FLT3, and c-Kit [10], [15], [16]. These may explain the broad preclinical activity of Sorafenib across tumor types and imply its clinical activity in anti-tumor treatment. Currently, Sorafenib has been approved for clinically use in hepatocellular carcinoma (HCC) and renal carcinoma. Notably, reported phase III studies showed a clear survival benefit in late stage HCC patients [14], [17]. Preclinical studies suggest that Sorafenib is also effective in other types of cancer cells such as non-small cell lung cancer and pancreatic cancer [10]. Both *in vivo* and *in vitro* studies suggest that Sorafenib inhibits tumor growth and disrupts tumor microvasculature through anti-proliferative and anti-angiogenic effects [8], [10], [14], [18]. Notably, angiogenesis is a very important factor for colon cancer growth [19]–[21]. Clinical studies also report the anti-tumor efficacy of Sorafenib in combination with other anti-cancer drugs, such as, irinotecan and rapamycin, for the treatment of colon cancer[2]. Therefore, it is promising to further develop Sorafenib derivatives which could enhance the anti-colon cancer effet of Sorafenib.

In this study, we are very interested to develop a new series of Sorafenib derivatives as a novel anti-colon cancer drug. The chemistry modification of Sorafenib has leaded to a new series of compounds with the enhanced antitumor activities and improved physiological properties. Our *in vivo* and *in vitro* screening of this series of Sorafenib derivatives shows HLC-080 (Figure 1) with an interesting *in vitro* anti-tumor activity. Therefore, HLC-080 is selected for further evaluation as a new anti-tumor candidate in colon cancer. We also tried to explain the possible mode of action of HLC-080 against colon cancer in order to support further drug development of Sorafenib derivatives being as novel anti-colon cancer drugs.

## Materials and Methods

### Materials

A novel diaryl urea derivative bearing sulfonamide moiety had been designed and synthesized based on the structure of sorafenib, which was named HLC-080. The synthetic procedure of HLC-080 was published in the journal of Sci China Chem [44]. Both HLC-080 and Sorafenib were prepared in our chemistry lab with the purity >97% (HPLC). For *in vitro* experiments, both compounds were dissolved in dimethyl sulfoxide (DMSO) and stored at 4°C for use. The vehicle (DMSO) was used as control in all experiments at a final concentration of 0.1%. For *in vivo* experiments, HLC-080 and Sorafenib were suspended in 0.5% carboxymethyl cellulose sodium (CMC-Na) in various concentrations.

### Cell culture

U251 and SHSY-5Y are human glioblastoma cell lines, HepG2, Bel7402 and SMMC-7721 are human hepatocellular carcinoma cell lines, HT-29, HCT-8 and HCT-116 are human colon cancer cell lines, MGC803 and BGC823 are human gastric carcinoma cell lines, A549 is human lung adenocarcinoma cell line, A2780 is human ovarian cancer cell line and HELF is human embryonic lung fibroblast. All of these cell lines were obtained from Institute of Basic Medical Science (IBMS) of CAMS & PUMC. Ketr3[39], [40] (human renal cell carcinoma) and EA.hy926 (human endothelial cell line) is purchased from American type culture collection (ATCC). All cells were cultured in DMEM or DMEM/F12 medium (Invitrogen) supplemented with 10% fetal bovine serum (Gibco, USA), 100 IU/mL penicillin, and 100 µg/mL streptomycin in a humidified incubator containing 5% CO_2_ at 37°C.

### Cell viability assay

2000 cells were seeded in a 96-well plate for 24 h. The cells were treated with various concentrations of compounds. After another 96 h of incubation, the MTT assay was performed to evaluate the cell viability. Each well was added with 200 µL of a 0.5 mg/mL solution of MTT (Sigma, USA) in incomplete medium. The plates were incubated in 37°C, 5% CO_2_ for another 4 h. The medium was carefully removed from each well and 200 µL of DMSO was added. The plates were gently mixed and read under microplate reader at OD570/OD450. Microsoft Excel 2010 was used for data analysis. Media-only treated cells served as the indicator of 100% cell viability. The 50% inhibitory concentration (IC_50_) was defined as the concentration that reduced the absorbance of the untreated wells by 50% as compared with the control cells. Each assay was performed in triplicate.

### Evaluation of anti-tumor effects in H22 xenograft model

CD-1 mice (males, 6-8 weeks old) were purchased from Vital River Laboratory Animal Technology Co., Ltd. (Beijing, China) and housed in controlled environment at 25°C on a 12-h light/dark cycle. All animal protocols are conformed to the Guidelines for the Care and Use of Laboratory Animals approved by the Animal Care and Use Committee of Chinese Academy of Medical Sciences and Peking Union Medical College. H22 (Murine liver cancer) cells were harvested and washed three times with normal saline. The cells were counted and diluted to 5×10^7^ cells/ml. CD-1 mice were subcutaneously implanted with 0.2 ml cell dilution on the right flank. Twenty-four hours after inoculation, CD-1 mice were randomly divided into 5 groups. Drugs were continuously administrated p.o. for 8 days, once a day. One group received p.o of 0.5% CMC-Na as a model control. Drug groups involved Sorafenib and HLC-080 at 0.15 mmol/kg and 0.30 mmol/kg, continuously were administrated p.o. for 8 days, once a day. At the end of the treatment period, the mice were dead and the tumors were excised and weighted. The inhibition rate (IR) of tumor growth was calculated by the following formula: IR (%)  =  [(A-B)/A]/100, where A is the average tumor weight of the model group, and B is that of the treatment group.

### Colony formation of colon cancer cells

Colon cancer cells were seeded in 6-well plate and diluted to 200 cells/well in cell culture media. After 24 h, cells were treated with different concentrations of drugs. After 14 days of incubation, colonies were produced and stained with Giemsa solution and counted each well. All samples were run in triplicate.

### Flow cytometry analysis

Cells were incubated with either Sorafenib, HLC-080 or DMSO (control) for 96 h. Then, the cells were washed twice with ice-cold phosphate buffered saline (PBS), harvested, fixed with ice-cold PBS in 70% ethanol, and stored at −20°C for 2 h. After fixation, the cells were incubated with RNase A (Sigma), stained with propidium iodide (Sigma) for 30 min in the dark. DNA content was analyzed by flow cytometric analysis.

### *In vitro* invasion assay

For the invasion assay, 1×10^6^ cells suspended in 1 ml of DMEM containing 0.1% BSA. The cells were seeded into the upper compartment (Costar) coated polycarbonate filter with a pore size of 8.0 µm in a 24-well plate. Each polycarbonate filter had been coated with 10 µL of 0.5% matrigel (Becton Dickinson, USA) before adding cells. DMEM medium (600 µL) containing 10% fetal bovine serum was added to the lower compartment as a chemo attractant. After 24 h of incubation at 37°C in 5% CO_2_, 90% relative humidity, the non-invasive cells presented on the upper surface of the filter were removed using a sterile cotton swab. The cells that migrated through the matrigel onto the lower surface of the filter were fixed and stained with HE stain at room temperature. The lower surface of the filter was photographed to compare the number of invasive cells. The five visual fields were photographed in every membrane (×200), The nuclear-stained cells were manually counted. All samples were run in triplicate.

### *In vitro* angiogenesis assay

The formation of capillary tube-like structures by EA.hy926 cells was analyzed on a 96-well cell culture plate coated with matrigel. The matrigel was thawed at 4°C overnight. 60 uL of pre-cooled matrigel was transferred to a 96-well plate and allowed it to solidify at 37°C for at least 1 h. Cells were seeded on the polymerized matrigel (3×10^5^ cells/ml). Dilute HLC-080 to be tested to required concentration in cell culture media. Incubate at 37°C, 5% CO_2_ for 6 to 24 h.

Endothelial cells tube size and numbers were observed under microscope and photographed for each well (×200). Any increase or decrease in the formation of tubes in the test wells as compared to control wells could be identified if the compound has an angiogenic effect or not.

### Western blot analysis

Cells were seeded and incubated at 5% CO_2_, 37°C overnight. Test compounds were added and incubated for another 2 h or 72 h at 5% CO_2_, 37°C. HT-29 cells total lysates were prepared by RIPA buffer (50 mM Tris pH 7.4, 150 mM NaCl, 1% Triton X-100, 0.5% deoxycholate, 0.1% sodium dodecyl sulfate) with protease inhibitor cocktails (Amresco, USA). Lysates containing 50 µg protein were used for Western blotting each sample. For western blot analysis, samples were separated on a 8%–10% SDS-PAGE gel, and transferred to a polyvinyldine diflouride membrane (Immobilon, Millipore, USA) by semi-wet electrophoresis. After blocking with TBST (0.1% Tween 20 in TBS) containing 5% nonfat milk, membranes were incubated with primary antibody [rabbit anti-p-c-Raf (Ser259), c-Raf, p-MEK1/2 (Ser217/221), MEK1/2, p-ERK1/2 (Thr202/Tyr204), ERK1/2, mouse anti-β-Actin] (CST) overnight at 4 °C. After three washings with TBST, the samples were detected with horseradish peroxidase-conjugated anti-rabbit or anti-mouse IgG (Santa Cruz), and developed using an ECL Western blot detection and analysis system (Applygen Technologies Inc.). Membranes were tested for equal loading by probing for actin.

### Enzyme-linked immunosorbent assay (ELISA)

Cells were seeded and incubated at 5% CO2, 37°C overnight. Different concentrations of test compounds were added and incubated for another 2 h at 5% CO2, 37°C. Whole-cell lysates of HT-29 cells were prepared by RIPA buffer, and subjected to ELISA analysis.

Microwell plates (COSTAR) were coated with capture antibody in coating buffer (100 µL/well). After overnight incubation at 4°C, the coated wells were washed three times with washing buffer and blocked at 37°C for 1 h by adding 100 µL of 2% BSA diluted in 0.1% Tween 20 in PBS. After a further washing procedure, 100 µL of standard dilutions and samples were added and the plate incubated for 1 h at 37°C. After washing three times, HRP-conjugated secondary antibody was added to the wells and the plates were incubated for 1 h exactly. After a final washing step bound antibodies were detected by the addition of 100 µL of TMB (Thermo 1-Step Ultra TMB). After 15 min the reaction was stopped with 100 µL 2 mol/L H_2_SO_4_ (sulphuric acid). Optical density (OD) was measured at 450 nm within 30 minutes.

### Statistical analysis

Data are expressed as the mean ± standard deviation. Statistical analysis of the results was performed using Student's t-test. P-values less than 0.05 were considered significant.

## Results

### Growth inhibition of HLC-080 and Sorafenib towards various cancer cells

The inhibitory effects of HLC-080 and Sorafenib on the proliferation of cancer cells were evaluated by MTT assay. In total, 13 types of cancer cell lines were tested (Table 1). In general, HLC-080 is more active against all the tested cancer cell lines compared to the mother compound Sorafenib. HLC-080 can inhibit the proliferation of Ketr3, HT-29, HCT-8, HCT-116, HepG2, SMMC-7721 at a moderate micromolar concentration, with IC50 values of 7.96, 1.83, 5.53, 5.66, 4.10, 7.48 µM respectively. Human colon cancer cells HT-29, HCT-8, HCT-116 were found to be more sensitive to HLC-080 than Sorafenib. Notably, the inhibitory effect of HLC-080 on HT-29 cell proliferation is about 5-fold higher than that of Sorafenib. Moreover, HLC-080 could inhibit the proliferation of HELF normal lung cells with IC50 values of 23.77 µM, which was 10-fold higher than that of colon cancer cells, indicating that HLC-080 was a selective inhibitor.

**Table 1:** The inhibition of HLC-080 and Sorafenib on the proliferation of various cancer cells.

Cell lines	Organs	IC_50_ (µM)	
		HLC-080	Sorafenib
U251	Brain	20.81±3.51	33.24±5.23
SH-SY5Y	Brain	45.36±5.24	32.70±5.81
MGC803	Stomach	15.68±3.95	20.83±4.18
BGC823	Stomach	26.94±4.75	37.26±5.41
Ketr3	Kidney	7.96±2.07	16.01±4.18
HT-29	Colon	1.83±0.35	9.37±1.18
HCT-8	Colon	5.53±1.29	18.27±3.52
HCT-116	Colon	5.66±0.96	12.93±2.89
HepG2	Liver	4.10±1.29	17.03±3.42
SMMC-7721	Liver	7.48±1.88	16.40±3.63
Bel7402	Liver	15.73±3.61	12.59±3.49
A549	Lung	27.64±4.77	29.60±4.61
A2780	Ovary	14.12±3.21	22.04±3.63
HELF	Normal lung	23.77±2.11	25.53±2.51

**Figure 2:**
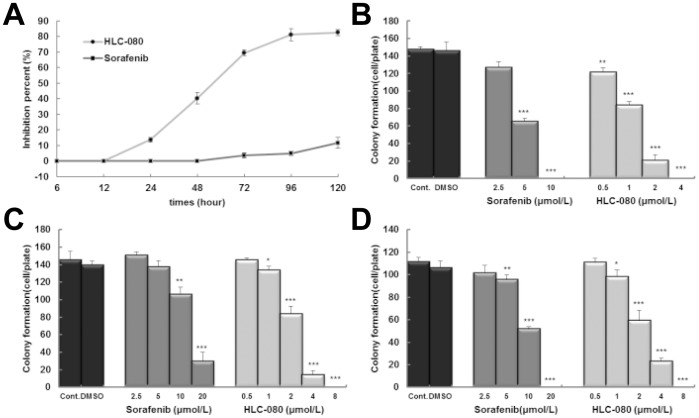
The anti-proliferation effect of HLC-080 in colon cancer cells . The time-effect relationship of 2.5 µM HLC-080 and 2.5 µM Sorafenib on proliferation in HT-29 cell line (A).The effect of HLC-080 and Sorafenib on colony formation in human colon cancer cell lines. HT-29 (B), HCT-8 (C) and HCT-116 (D) cells were seeded in a 6-well plate. After 24 h, cells were treated with various concentrations of HLC-080 or Sorafenib. After 14 days of incubation, observe colony size and numbers by Giemsa stain and photograph each well. * P<0.05, ** P<0.01, *** P<0.001, compared with control group.

In addition, both HLC-080 and Sorafenib show time-dependent effect on cell proliferation in HT-29 cell line (Figure 2A). After 12 h of the incubation, 2.5 µM HLC-080 is constantly more active against the HT-29 cells than the same dose of Sorafenib at all measured time points.

On the other hand, we confirm the higher anti-proliferation effect of HLC-080 in colon cancer cells than Sorafenib by using colony formation experiments. Results shows EC_50_ of Sorafenib on HT-29, HCT-8, HCT-116 cell lines were 3.96, 11.87 and 11.23 µM, respectively. In contrast, the EC_50_ to HLC-080 were 0.91, 2.21 and 2.23 µM respectively (Figure 2B). Therefore, HLC-080 exhibits around 4 to 5 folds more active antitumor effects in colon cancer cell lines.

### *In vivo* antitumor activity of HLC-080 and Sorafenib in mice H22 xenograft model

Since HLC-080 and Sorafenib showed anti-proliferation activities against colon cancer cell lines from *in vitro* studies, we further screened their *in vivo* anti-tumor activities in mice. H22 HCC xenograft model was employed to evaluate the general *in vivo* anti-tumor activity of our compound HLC-080. The facility and stability of the H22 tumor model made it one of the most commonest used tumor models in anti-tumor research [28], [29]. Our results presented that both HLC-080 and Sorafenib could inhibit the tumor growth on H22 xenografts model. Both of them caused tumor regression compared to the control group, as shown in Table 2.

**Table 2:** The effect of HLC-080 and Sorafenib in H22 xenograft model *in vivo*.

Group	Dose (mmol/kg)	No. of animals(n)	Body Weight (g)		Tumor weight (g)	IR (%)
		Begin/End	Begin	End		
Control		10/10	18.16±0.79	27.33±1.87	3.09±0.42	
Sorafenib	0.15×4d+0.075×4d	10/10	17.73±0.47	26.29±2.18	1.28±0.40***	58.63
	0.30×4d+0.15×4d	10/5	17.86±0.67	25.02±3.02	1.18±0.45***	61.84
HLC-080	0.15×8d	6/6	18.00±0.54	25.85±2.07	1.98±0.46***	35.81
	0.30×8d	6/6	17.31±0.56	26.56±2.54	2.07±0.59***	33.04

*** P<0.001, compared with control.

Some side effects were observed in Sorafenib group, including anorexia, unkempt appearance, weight loss or even death. Therefore, for the rest of the time the dosage of two Sorafenib groups was reduced to half of the initial dosage. In comparison, although animals in two HLC-080 groups also showed unkempt appearance and weight loss, none of them were dead during the treatment period.

Compared with the control group, the mice treated with 95 and 190 mg/kg of Sorafenib group inhibited tumor growth. The inhibitory rates at the end of the treatment were 58.63% and 61.84% (Table 2, P<0.001), respectively. On the other hand, for the groups upon HLC-080 treatment with 75 and 150 mg/kg, the inhibitory rates were 35.80% and 33.04% respectively. All these data suggested that HLC-080 has satisfactory inhibition effect against H22 tumor growth but with fewer side effects comparing to Sorafenib.

### Cell cycle arrest by HLC-080

**Figure 3:**
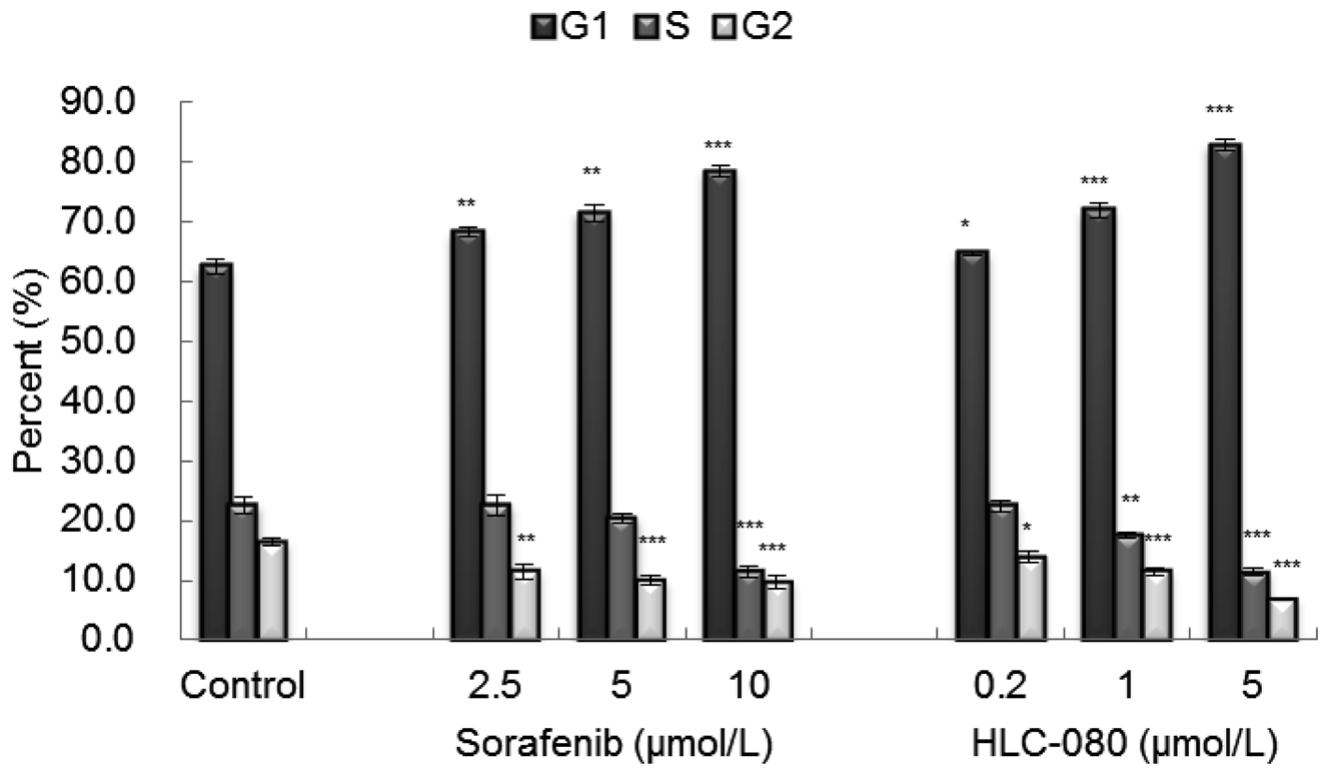
Sorafenib and HLC-080 induced cell cycle arrest at G1 phase. Cells were incubated with various concentrations of Sorafenib and HLC-080 for 96 h, cells were then harvested and stained with DAPI and analyzed by flow cytometry. Error bars indicate standard deviation. *p<0.05, **p<0.01, ***p<0.001, compared with control group.

HLC-080 can inhibit the proliferation of HT-29, HCT-8 and HCT-116 colon cancer cells. Subsequently, we analyzed the cell cycle distribution of the HT-29 cells treated with either HLC-080 or Sorfenib using Flow cytometry. As shown in Figure 3, after treatment with 2.5, 5, 10 µM Sorafenib and 0.2, 1, 5 µM HLC-080 for 96 h, Sorafenib and HLC-080 exhibited an increase in the proportion of cells in G0–G1 phase in a dose dependant manner, correspondingly with a decrease in the proportion of the cells in S and G2/M phase. These results suggested that the proliferative inhibition of HLC-080 was partially due to a G1-phase arrest in HT-29 colon cancer cells.

### The effect of HLC-080 on vascular tube formation

Angiogenesis is a crucial factor for colon cancer growth. One of the most widely used *in vitro* assay to model the formation of angiogenesis is known as the tube formation assay [22]. HLC-080 was evaluated in the tube formation model in order to investigate its anti-angiogenic effect.

**Figure 4:**
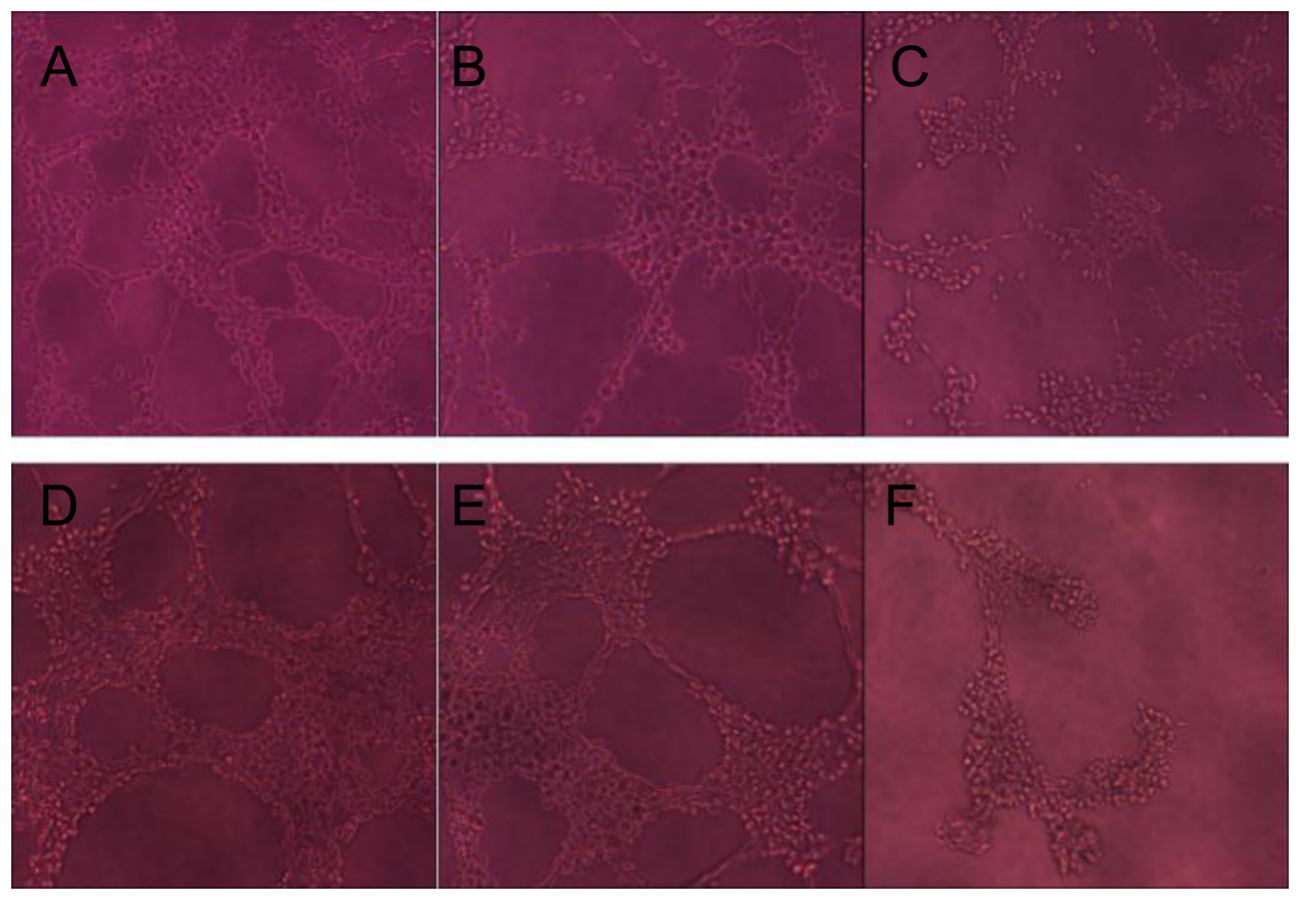
The effect of HLC-080 on EA.hy926 vascular tube formation. EA.hy926 were seeded on matrigel in 96-well plates at 3×10^4^ cells per well. The cells were mixed with different concentrations of HLC-080 and photographed at various time points, as described in the methods. (A) Control at 6 h showing EA.hy926 without additional stimuli. (B) HLC-080 1.0 µM added to EA.hy926, showing vascular tubes at 6 h. (C) HLC-080 5.0 µM added to EA.hy926, showing vascular tubes at 6 h. (D) Control at 24 h showing EA.hy926 without additional stimuli. (E) HLC-080 1.0 µM added to EA.hy926 formed only a small number of short, incomplete tubes at 24 h (F) HLC-080 5.0 µM added to EA.hy926, exhibited more significant effects at 24 h.

We first tested the growth inhibition of HLC-080 on EA.hy926 cells in order to dose properly in the vascular tube formation experiment. The IC_50_ for HLC-080 against EA.hy926 is 29.6±3.67 µM at 24 h. We observed the vascular tube formation between 6 h to 24 h after plating as during this period the vascular tube forming process reached a maximum. We found that in the group of HLC-080 1.0 µM, EA.hy926 only formed a small number of short and incomplete tubes. At the same time point, 5.0 µM HLC-080 exhibited a significant effect on ***. After 24 h, the tubes began to disintegrate. In general, remarkable visual differences were observed between HLC-080 treated groups and the untreated groups (Figure 4).

### Reduction in the invasive potential of HT-29 colon cancer cells by HLC-080

**Figure 5:**
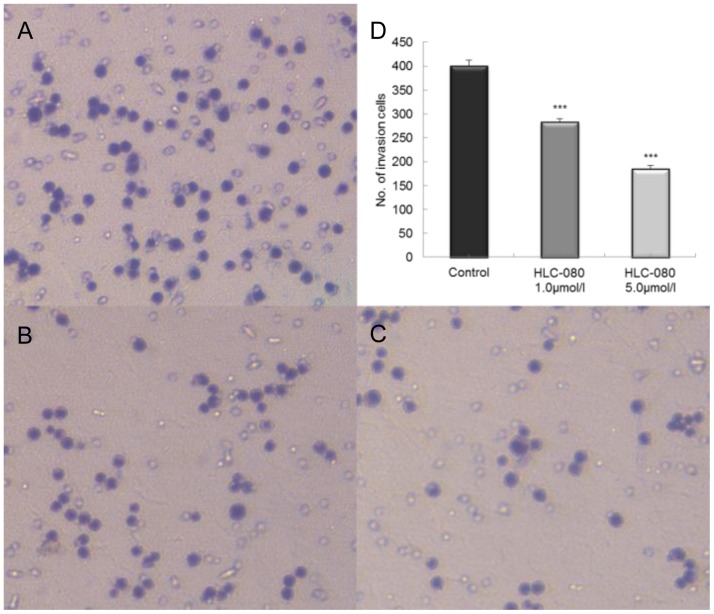
The effect of HLC-080 on cell invasion in HT-29 cells. Cells were seeded in a transwell chamber and allowed to migrate across the chamber toward cell-specific conditioned medium for 24 h. Photomicrographies of stained migrating cells were taken under brightfield illumination. Representative images are shown for HT-29 control (A) and HT-29 disposed by HLC-080 1.0, 5.0 µM (B, C). Quantification of the invasion is expressed as the number of invasive cells in five random microscopic fields per well (mean ± SD; ***p<0.001 versus control group cells) (D). Results were obtained from three separate experiments.

We investigated whether HLC-080 could reduce the HT-29 cell invasion using transwell assays. As shown in Figure 5, 1.0 µM and 5.0 µM of HLC-080 reduced the invasion ability in HT-29 cells, at the inhibition rate of 29.23% and 53.71%, respectively, compared with the control group. These data showed that HLC-080 does affect the invasive potential of colon cancer cells *in vitro*.

### The mode of actions of HLC-080 in HT-29 cell line

Raf kinases are well-known as key regulators of the MEK/ERK cascade, and up-regulated signaling through the Raf/MEK/ERK pathway has an important role in various cancers [41], [42], [43]. In order to find out the mechanism responding for growth inhibition by HLC-080, we examined the expression level of Raf/MEK/ERK in HT-29 cell line by western blot. To detect the p-c-Raf (Ser259) expression in colon cancer cells, we performed the ELISA experiment. Both ELISA and western blot confirmed the presence of p-c-Raf (Ser259) in untreated HT-29 cells.

**Figure 6:**
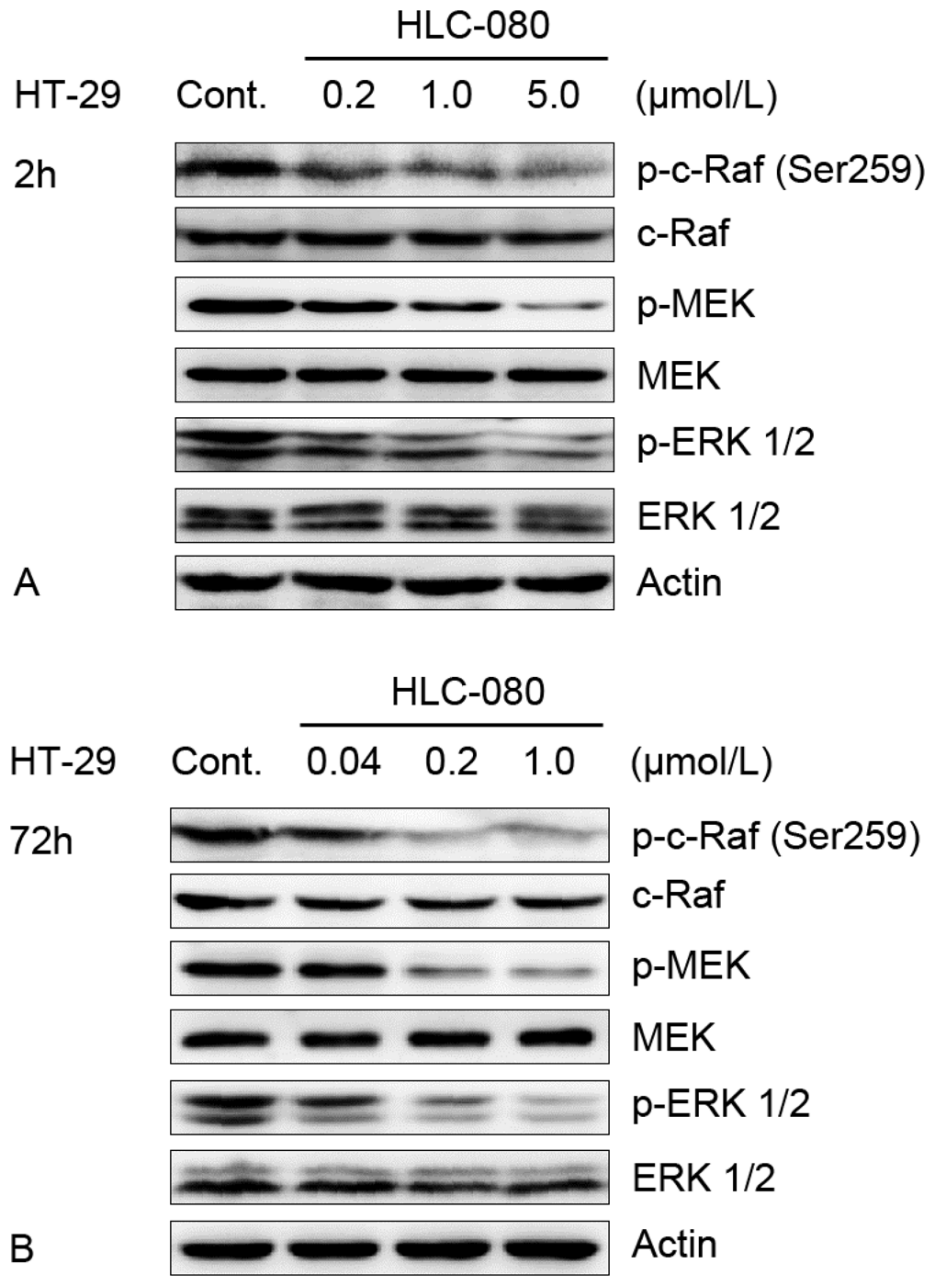
The effect of inhibition of Raf/MEK/ERK pathway in HT-29 cells *in vitro*. (A) HT-29 cells were incubated with increasing concentrations of HLC-080 for 2 h. (B) HT-29 cells were incubated with increasing concentrations of HLC-080 for 72 h. Whole-cell lysates were subjected to western blot analysis. For western blot analysis, samples were transferred to a polyvinyldine diflouride membrane by semi-wet electrophoresis and incubated with indicated primary antibody p-c-Raf (Ser259), c-Raf, p-MEK1/2 (Ser217/221), MEK1/2, p-ERK1/2 (Thr202/Tyr204), ERK1/2). Actin was used as loading and transfer control. The experiment was repeated twice and similar results were obtained.

Figure 6A showed that both 1.0 µM and 5.0 µM of HLC-080 started to inhibit the p-c-Raf (Ser259) and p-MEK (Ser217/221) in HT-29 as early as at 2 h. After 72 h treatment with 0.2 µM and 1.0 µM HLC-080the levels of p-c-Raf Ser259, p-MEK1/2 (Ser217/221) and p-ERK1/2 (Thr202/Tyr204) remarkably decreased in HT-29 cells (Figure 6B). On the other hand, we found that 0.2 µM HLC-080 became more active against the HT-29 cells when the treatment time was elongated from 2 h to 72 h.

We confirmed the effect of HLC-080 on key mediators in the Ras/Raf/MEK/ERK signaling pathway. To better understand how HLC-080 acted inside the cells, we also tested the inhibitory activity of them towards tyrosine kinase with Sorafenib used as a positive control.

HLC-080 inhibited the p-c-Raf (Ser259) in the sub-micromolar concentration, with EC_50_ values of 0.357 µM which was about 3-fold higher than that of Sorafenib (Table 3). This suggests that p-c-Raf might be the primary targets of HLC-080 inside the cells so that these anti-proliferation effects could be achieved.

**Table 3:** HLC-080 and Sorfenib inhibits p-c-Raf (Ser259) activities *in vitro*.

Compound	EC_50_ (µM)
	HT-29
HLC-080	0.352±0.034
Sorafenib	0.957±0.039

## Discussion

Colon cancer is one of the most common cancers in the world [1], [3], and it has been estimated that 50,830 patients died from colon cancer in the United States in 2013 [1]. Current first line drugs for colon cancer chemotherapy cannot achieve a satisfing clinical result. In order to improve the quality of colon cancer chemotherapy, there is a need to call for more specific and effective drugs for the treatment of colon cancer.

Preclinical studies suggest that Sorafenib, a known oral anticancer drug, can inhibit cancer cell proliferation and angiogenesis and subsequently acts on tumors and tumor vasculature [10], [23]. It is worth trying to apply Sorafenib as anti-colon cancer drugs. However, the pharmacological effect of Sorafenib and the targeted signaling pathway in colon cancer is not yet fully elucidated. Also, it is necessary to abolish or reduce the drug related-side effect of Sorfenib, such as, pruritus, rash/desquamation and hypertension [18], [24]–[27], before its clinical usage for the colon cancer treatment.

We are attempting to develop a novel Sorafenib derivative as an anti-colon cancer drug with fewer side-effects. The chemical modification of Sorafenib has generated a new series of compounds with promising antitumor activities. One of them, HLC-080 was discovered and selected, to evaluate the effect of these Sorafenib derivatives on the inhibition of tumor cell growth and colony formation.

Among 13 types of human cancer cells, HT-29, HCT-8, HCT-116 colon cancer cells were found to be more sensitive to HLC-080 than Sorafenib. Particularly, HLC-080 exhibited a stronger inhibitory effect against HT-29 colon cancer cells than Sorafenib with a time dependant manner.

H22 HCC xenograft model was employed to evaluate the general *in vivo* anti-tumor activity of our compound HLC-080, One advantage of this model is that the ascitic-type liver cancer H22 can grow in many mouse strains with high invasiveness and metastasis. The facility and stability of the H22 tumor model made it one of the most commonest used tumor models in anti-tumor research [28], [29].

In the previous study using the HCC xenograft model, 100 mg/kg Sorafenib could significantly reduced tumor growthwith partial tumor regressions were observed in 50% of the mice [10], [30]. Our results from Sorafenib are in agreement with those findings. HLC-080, on the other hand, not only inhibits tumor volume and weight but also demonstrates more significant effect than Sorafenib at the same dose (0.15 mmol/kg)… Sorafenib is hard to be absorbed in the gastrointestinal tract due to its poor water solubility [31], and HLC-080 has the same problem. For *in vivo* experiments, different concentrations of HLC-080 and Sorafenib were suspended in 0.5% CMC-Na, but all of them were hard to achieve a decent dissolution rate. We anticipate that the improved solubility and dissolution of HLC-080 in the salt formation will increase drug absorption and therefore enhances antitumor activities *in vivo*.

Based on *in vitro* and *in vivo* anti-tumor activity of HLC-080 provided in our experiments, we further conducted experiments to understand the possible mode of action of HLC-080 for the treatment of colon cancer. Angiogenesis is essential for solid tumor growth and metastasis. As a result, anti-angiogenesis became an attractive way against tumor [32]-[34]. We examined the anti-angiogenic effect of HLC-080 *in vitro* by observing, the tube formation in EA.hy926 cells A marked decrease in the area of tube growth was observed with only 1.0 uM of HLC-080.. Providing that low concentration of HLC-080 exhibits little cytotoxic effect, HLC-080 can serve as a safe angiogenesis inhibitor.

According to recent reports [13], [14], [30], [35], 5∼10 µM of Sorafenib had been shown to be a therapeutically achievable concentrations in preclinical trials. Our results support those findings. Our studies revealed that both 1.0 µM and 5.0 µM of HLC-080 can induce cell cycle arrest at G1 phase in HT-29 cells. At the same time, Sorafenib can regulate the cell cycle and decrease cell proliferation at 5.0 and 10 µM respectively. Since ERK is required for G1/S transition through many aspects [36], we speculate that decreased level of p-ERK by HLC-080 may be attributable to cell cycle arrest at G1 phase in HT-29 cells.

Previous study shows that more than 50% of colon cancer patients have metastatic and invasive tumor, so the treatment of invasive colon cancer faces various challenges. [3] The treatment of metastatic and invasive colon cancer usually involves chemotherapy or combination therapy [2]. The results in our present study indicates that 5.0 µM HLC-080 can reduce the invasion ability in HT-29 cells, at the inhibition rate of 53.71%. It has been reported that specific blockage of ERK pathway in colon tumor cells could inhibit the cross-talk and motility which are required for metastasis and invasion [37]. Our results support those findings. Raf isoforms are on the top of Raf/MEK/ERK signaling and can activate serine/threonine kinase. Raf induced phosphorylation of MEK which in turn phosphorylates ERK/MAPKs. In this study, we show that HLC-080 inhibited the phosphorylation of c-Raf and therefore decrease the level of p-MEK and p-ERK. Some reports have suggested that in colon tumors, tumor growth inhibition correlated with a decrease in ERK phosphorylation [10], [14]. And p-ERK as a biomarker support a role for Raf inhibition in the mechanism of action of Sorafenib in both HCC and colon cancer [7], [30]. According to recent report, 10∼20 µM of Sorafenib had been shown to decrease the level of p-ERK in HT-29 cells [38] Means that HLC-080 have a stronger inhibition effect than Sorafenib in colon cancer cells. Our findings suggested that inhibition of cell proliferation via Raf/MEK/ERK signaling pathway might be one of the mechanisms contributing to the effects of HLC-080 in colon cancer.

Our study presented that HLC-080 is able to inhibit p-c-Raf (Ser259) with an EC_50_ of 0.352±0.034 µM. This finding supports that the biological effect of HLC-080 on HT-29 is related to the suppression of the Raf/MEK/ERK pathways. However, in this study the inhibition of HLC-080 towards p-c-Raf (Ser259) was evaluated using whole-cell lysates, the inhibition effect of HLC-080 can be further validated at the enzyme level.

In conclusion, this study describes the biochemical and pharmacological activities of HLC-080, an inhibitor of Raf kinases with potential to block proliferation, inhibit angiogenesis and induce cell cycle arrest as well as prevent the invasion in colon cancer cells. The further analysis unveils that such effects might be induced by blocking the Raf/MEK/ERK signaling pathways. Our findings highlight the promising utility of HLC-080 for colon cancer treatment, though the further investigation of HLC-080 in pre-clinical and/or clinical setting is requested.
